# Presentation Patterns, Diagnostic Modalities, Management Strategies, and Clinical Outcomes in Patients with Hydatid Disease of the Pelvic Bone: A Comparative Review of 31 Cases

**DOI:** 10.7759/cureus.4178

**Published:** 2019-03-05

**Authors:** Faisal Inayat, Shoaib Azam, Areeba S Baig, Gul Nawaz, Waqas Ullah

**Affiliations:** 1 Internal Medicine, Allama Iqbal Medical College, Lahore, PAK; 2 Orthopaedics, King Edward Medical University, Lahore, PAK; 3 Internal Medicine, King Edward Medical University, Lahore, PAK; 4 Internal Medicine, Allama Iqbal Medical College, Lahore , PAK; 5 Internal Medicine, Abington Hospital-Jefferson Health, Abington, USA

**Keywords:** hydatid disease, pelvic bone, epidemiology, clinical presentation, lesion site, diagnosis, management, clinical outcomes

## Abstract

Hydatid disease is a parasitic zoonosis commonly caused by *Echinococcus granulosus*. It characteristically involves the liver and the lungs but rare occurrences in other organs have also been reported. Bone involvement is distinctly uncommon, which is predominantly a silent and slowly progressive disease with a long latent period. We conducted a systematic literature search of MEDLINE, Cochrane, Embase, and Scopus databases. After a comprehensive review of the search results, a total of 31 cases of hydatid disease of the pelvic bone fulfilled the inclusion criteria. The data on patient demographics, epidemiology, lesion site, management, clinical outcomes, and follow-up were collected and analyzed. This review illustrates that hydatid disease should be considered among the differential diagnoses of unusual cystic lesions of the pelvic bone. Prompt diagnosis and appropriate management are of paramount importance to prevent bone destruction and serious complications in these patients. Long-term follow-up should be performed for potential recurrence.

## Introduction and background

Human hydatid disease, also known as cystic echinococcosis, is most commonly caused by *Echinococcus granulosus*. The lesions of this infectious etiology can be encountered in myriad body locations. While it frequently involves the liver and the lungs, bone involvement is exceedingly rare [1, 2]. Although the incidence of bone disease is remarkably low, its diagnosis and management can be challenging. The clinical presentation of patients with osseous hydatid disease is frequently nonspecific. Occasionally, pain and pathological fractures are the presenting symptoms [3, 4]. In regard to the diagnosis, the findings of clinical history, laboratory studies, radiologic investigations, and histopathologic analysis play a key role [5, 6]. Surgical resection of the cystic lesions with antihelminthic chemotherapy is the treatment of choice. Bone curettage, achieving the negative resection margins, is essentially important to avoid the recurrence of the disease [7]. The prognosis is usually favorable but early treatment may save the patients from inadvertent events as well as excessive surgical debridement requiring bone replacement [8]. The aim of this comparative review was to summarize the data on clinical presentations, diagnostic strategies, management options, and the clinical outcomes in patients with pelvic bone hydatidosis. This paper emphasizes that the clinicians should be vigilant for this disease, particularly in patients presenting with nonspecific skeletal symptoms.

## Review

Materials and methods

We conducted a systematic literature search to retrieve published data on pelvic bone hydatidosis using the MEDLINE, Cochrane, Embase, and Scopus databases. Several controlled vocabulary search terms (medical subject headings [MeSH] and Embase subject headings [Emtree]), terminologies like “hydatid disease,” “bone,” “pelvis,” “pelvic involvement,” and “management” were combined using the Boolean operators “AND” and “OR” with the terms “hydatidosis,” “outcome,” and “chemotherapy.” The search was conducted without a defined time filter, with language limitation to English-only articles. Additionally, a manual search was also performed using the bibliography of all accessed publications through the above-mentioned search strategy. We initially screened all retrieved titles and abstracts to determine their relevance to our topic. The same protocol was used to screen the selected articles for full texts to check their relevance.

Results

A total of 127 studies were initially obtained, consisting of but not limited to original articles, case series, and case reports. After excluding 49 duplicate articles, 78 papers were thoroughly studied. The articles available in any language other than the English were excluded from the review. Thirty-two papers were found relevant to the scope of our study but 15 studies were found accessible in order to retrieve pertinent data required for this review [9-23]. The total number of patients in this comparative review was 31 as some case studies consisted of more than one patients. The data on individual cases of pelvic bone hydatidosis on patient demographics, locations of the lesions, management, and clinical outcomes are summarized (Table 1).

**Table 1:** The Demographic, Lesion Site, Treatment, and Outcome Data of Patients with Pelvic Bone Hydatidosis.

Authors	Publication year	Country	Age/gender	Lesion site	Management	Outcome
Agarwal et al. [9]	1991	KSA	70/F	Ilium and sacroiliac joint	Surgical excision and chemotherapy	Symptom-free in 18-month follow-up
Agarwal et al. [9]	1991	KSA	43/F	Pubis, ischium, acetabulum, and proximal femur	Surgical excision and chemotherapy	Symptom-free in four-year follow-up
Wirbel et al. [10]	1995	Yugoslavia	49/M	Ilium, pubis, acetabulum, and proximal femur	Chemotherapy for five years followed by partial pelvic resection and prosthetic replacement	Recurrence was treated with custom-made prosthesis
Belzunegui et al. [11]	1997	Spain	54/F	Hemipelvis and proximal femur	Girdlestone arthroplasty and chemotherapy	Recurrence was managed with chemotherapy
Martinez et al. [12]	2001	Spain	56/M	Ilium and sacral ala	One surgical drainage and chemotherapy	Symptom-free in three-year follow-up
Martinez et al. [12]	2001	Spain	62/F	Ilium	Two surgical drainages and chemotherapy	Symptom-free in six-year follow-up
Martinez et al. [12]	2001	Spain	58/F	Ilium	One surgical drainage and chemotherapy	Symptom-free in four-year follow-up
Martinez et al. [12]	2001	Spain	64/F	Ilium	One surgical drainage and chemotherapy	Symptom-free during five-year follow-up
Martinez et al. [12]	2001	Spain	47/M	Ilium and sacral ala	Three surgical drainages and chemotherapy	Symptom-free in seven-year follow-up
Martinez et al. [12]	2001	Spain	68/M	Ilium and hip	Numerous surgical drainages and chemotherapy	Non-functioning limb, productive sinuses, hip pain, walking difficulty in 13 years of follow-up
Martinez et al. [12]	2001	Spain	76/M	Ilium and hip	Numerous surgical drainages and chemotherapy	Non-functioning limb, productive sinuses, hip pain, walking difficulty in nine-year follow-up
Martinez et al. [12]	2001	Spain	74/M	Ilium and hip	Three surgical drainages and chemotherapy	Non-functioning limb, hip pain, walking difficulty in 12-year follow-up
Masse et al. [13]	2004	Italy	25/M	Ischium	Five local excisions of the cyst and chemotherapy	Symptom-free during 12 years of follow-up
Khan et al. [14]	2008	Nepal	41/M	Superior pubic ramus and body	Antihelminthic chemotherapy	Symptomatic improvement after three months
Siwach et al. [15]	2009	India	51/F	Femur (pathological fracture) and pelvis	Hindquarter amputation and chemotherapy	Death due to sepsis
Nath et al. [16]	2009	India	35/M	Ilium, acetabulum, ischial tuberosity, and pubic rami	Wide excision	Not reported
Winning et al. [17]	2009	Australia	77/F	Left femur, hemipelvis, and adjacent soft tissues	Femoral head excision and chemotherapy	Septic shock likely from secondary bacterial infection of the hip sinus
Neelapala et al. [18]	2010	UK	35/F	Hip joint and ileum	Chemotherapy for two years followed by cemented total hip replacement	Recurrence that required customized hemipelvic replacement
Notarnicola et al. [19]	2010	Italy	53/F	Proximal femur (pathological fracture)	Total hip replacement	Recurrent dislocation and disassembly; revised with Wagner-type prosthesis
Liang et al. [20]	2014	China	29/M	Ilium, ischium, pubis, hip, and greater trochanter	One debridement, hemipelvis replantation with femoral prosthesis replacement, and chemotherapy	Symptom-free in seven years of follow-up
Liang et al. [20]	2014	China	26/F	Ilium	Four debridements and chemotherapy	Symptom-free in nine years of follow-up
Liang et al. [20]	2014	China	24/F	Ilium and acetabulum	Two debridements, bone cement filling, and chemotherapy	Symptom-free in five-year follow-up
Liang et al. [20]	2014	China	45/F	Acetabulum	Internal fixation with bone cement filling	Symptom-free during two years of follow-up
Liang et al. [20]	2014	China	31/M	Ilium	Two debridements with radiotherapy and chemotherapy	Symptom-free in six years of follow-up
Liang et al. [20]	2014	China	52/M	Ilium	Numerous debridements	Hip pain during seven years of follow-up
Liang et al. [20]	2014	China	14/F	Ilium and sacrum	Numerous debridements, one screw and rod fixation, and chemotherapy	Walking difficulty, productive sinuses during 19 years of follow-up
Liang et al. [20]	2014	China	35/F	Ilium and acetabulum	Five debridements and chemotherapy	Hip pain during seven years of follow-up
Liang et al. [20]	2014	China	32/F	Ilium and sacroiliac joint	Three debridements with radiotherapy, and chemotherapy	Walking difficulty in four years of follow-up
Raj and Dash [21]	2015	India	40/M	Ilium, ischium, pubis, sacrum, hip joint, and head and neck of femur	Wide local excision, curettage, internal fixation of the right femur and chemotherapy	Disease-free and ambulant with crutches
Tsagozis and Brosjo [22]	2015	Turkey	56/M	Hemipelvis, sacrum, femur	Extended hemipelvectomy	Symptom-free at one-year follow-up
Bhatnagar et al. [23]	2017	India	35/F	Femoral head and acetabulum	Surgical debridement and chemotherapy	Disease-free after three months

A comprehensive review of these cases indicated a slight female predominance (female, n = 17; male, n = 14). The mean age of patients was 47 years (range: 14-77 years). According to our analysis, ilium (n = 21) was the most frequent lesion site followed by acetabulum (n = 7), pubis (n = 6), ischium (n = 5) and sacrum (n = 5). In a few patients, concurrent involvement of the long bones like femur and/or soft-tissue structures of the pelvis was also noticed.

Epidemiology

According to one estimate, the incidence of bone involvement has been described to range from 0.5% to 4% of all cases of hydatidosis [24]. The bone disease predominantly involves the spine, which is encountered in approximately 50% to 60% of the cases, followed by the femur, tibia, humerus, skull, and ribs. With regard to geographical distribution, hydatid disease is globally distributed, especially in the north and northwestern China, parts of South America, East Africa, Australia, Central Asia, North Africa, Russia, Western Europe, and southern United States [24]. The highest prevalence is noted in rural areas where animals are slaughtered. In the recent past, several hydatid elimination programmes had been implemented, with varying degrees of success. Recently, hydatid vaccine has also been considered to combat this crippling infestation.

Pathogenesis

Although the bone is an uncommon location for hematogenous dissemination of hydatid disease, the presence of the lesions has frequently been reported. The progression of the disease takes place either due to the formation of diverticula or exogenous vesiculation. This disease ensues destructive pathological osseous sequels predominantly by three mechanisms: a) the cyst that increases in size gradually compresses the adjacent tissues, eventually causing compression-related bone atrophy, b) occasionally, the hydatid cyst may obstruct the vessels entering the bones through nutrient foramina causing ischemia, c) the cells like osteoclasts, proliferate around the infectious focus of hydatidosis. On the contrary, the extraosseous invasion culminates in the bone disruption that subsequently may lead to pathologic fracture of the involved portions of the bone [24].

Clinical presentation

The clinical presentation of bone hydatidosis is frequently varied. It is notable that the bone involvement is largely a silent disorder that has the propensity to remain dormant for decades. Pain, swelling, walking abnormalities, sinus tract formation, ambiguous abdominal pain, or compressive manifestations are among the symptoms and signs of this disease. In patients with hydatidosis of pelvis involving the lumbosacral neural plexus, sciatica becomes the first clinical symptom. Similarly, a vast majority of patients initially present with late complications of this disease like a pathologic fracture, neurological deficits, infected cyst or fistula formation [24]. The physical examination is mostly inconclusive for abnormalities. However, in rare instances, changes in the limb symmetry, abscess or fistula formation or vertebral deformation can be deciphered. Individuals like sheepherders, veterinarians, or butchers are particularly prone to this infection. Therefore, this disease warrants awareness and updated knowledge on part of clinicians, especially in endemic areas when patients present with generalized musculoskeletal complaints.

Diagnosis

The diagnosis of pelvic bone hydatidosis frequently poses a challenge as pelvic localization by radiological modalities may be difficult and clinical features are mostly nonspecific. Although serological testing has gained importance recently, their sensitivity and specificity is not 100% [25]. Computed tomography is an excellent modality to detect osseous hydatidosis. The radiologic investigations often demonstrate multiple expanding lesions with no defined margins. The lesions may assume a classic waffle appearance due to extensive osteolysis [26]. The unique pathogenetic changes encountered in this disease lead to weakening of the cortical bone, without any periosteal reaction. Magnetic resonance imaging has also been used to investigate the regional disease extent, especially soft-tissue involvement. The hydatid lesions appear as a hyposignal in T1-weighted images and a hypersignal in T2-weighted images [27]. In these patients, a whole-body scan is performed in order to assess the concurrent involvement of other organ systems. As the diagnostic dilemma is frequently noted, tuberculosis, malignancy, aneurysms, and metastatic lesions should be excluded based on the standard set of investigations [27]. A definitive diagnosis is established on the basis of histopathologic examination of the biopsy and/or resected specimen.

Management

In terms of management, hydatid disease of the pelvic bone is particularly a serious clinicopathologic entity as the cyst in this location may invade pelvic joints, which can potentially make complete recovery difficult. Although the definitive treatment of bony hydatidosis is surgery, a number of studies have highlighted the combination of antihelminthic chemotherapy and surgery as a feasible choice [28]. In the published medical literature, several surgical methods, including simple drainage or debridement, complete excision, total hip arthroplasty, bone grafting, femoropubic arthrodesis, megaprosthetic replacement, massive arthroplasty, osteosynthesis, and hemipelvectomy have been reported thus far [29].

The procedure of simple drainage or debridement is commonly employed; however, early recurrence and inadvertent disease dissemination may occur due to incomplete removal. In this technique, the lesion is exposed while adjoining normal structures are protected with the use of 20% NaCl solution. The burnishing of the inner walls of the lesion cavity is also important to avoid recurrence of the disease. This method is mostly used in patients where cystic echinococcosis cannot be excluded preoperatively [29]. Furthermore, bone cement filling is a reliable option to avoid the relapse of the cystic lesions due to its ability to kill the daughter cysts due to necrotizing effects of increased temperature in the polymerizing cement [30]. Several surgical methods are used for reconstruction of osseous portions affected by the hydatidosis. A pedicle screw-rod system is an effective reconstructive option with no need for extensive preoperative feasibility assessment but it may present a dilemma while treating joint involvement. Partial excision of the cystic lesions followed by joint arthroplasty is a good method in this regard [18]. Similarly, megaprosthetic replacement may also help to restore acceptable limb functionality [10]. The major goal of these procedures is to restore the limb function rather than complete eradication of the infectious etiology due to echinococcosis. The use of liquid nitrogen carries several benefits; however, its role in disinfection of bony *Echinococcus* cysts has rarely been documented. However, Liang et al. supported the notion that bony cystic lesions due to *E. granulosus* maybe enervated by utilizing liquid nitrogen for a time period of 20 minutes [20].

The therapeutic strategy for hydatid disease with bone involvement resembles oncologic therapy compared to the surgical treatment of visceral hydatidosis [24]. In these patients, a combination of preoperative antihelminthic chemotherapy, surgery, and postoperative antihelminthic chemotherapy demonstrates promising outcomes. Before surgical intervention, medical therapy, mostly consisting of albendazole, targets the cyst size reduction and limits the infectious process. In the post-operative setting, it is primarily used for the treatment of potentially undetectable cysts, ultimately avoiding the recurrence. Similarly, irradiation can be used in patients who cannot tolerate the chemotherapy and/or in inoperable disease [31].

Prognosis

The prognosis of hydatid disease of the pelvic bone largely depends on its bony extensions. In cases with a widespread disease demonstrating an involvement of the pelvic joints and long bones, the prognosis is generally poor. These patients are particularly prone to life-threatening sepsis. Furthermore, if the disease involves several muscle groups or muscle layers, the prognosis is not good and it poses a significant therapeutic challenge due to the presence of sinus and/or fistula formation [32]. In the light of these observations, early diagnosis of pelvic bone hydatidosis is critically important as a late detection makes it a difficult-to-treat disease [33].

## Conclusions

Although pelvic bone hydatidosis is rare, a high index of clinical suspicion should be maintained for this disease, especially in endemic areas. These patients may pose a diagnostic challenge due to nonspecific clinical presentation. The radiological characteristics of the hydatid cyst often suggest the pathology but a definitive diagnosis can only be made by histopathologic examination. Early diagnosis is of paramount importance for the bone salvage and to avoid complications. The treatment of choice is a combination of chemotherapy and surgical debridement. Meticulous technical preparation is necessary as surgery of the pelvis is relatively difficult and incomplete resection of the cyst may culminate in recurrence. The present paper highlights the importance of early detection of the pelvic bone hydatidosis followed by efficient management for a good clinical outcome.
